# β-Defensin 2, an Antimicrobial Peptide, as a Novel Biomarker for Ulcerative Interstitial Cystitis; Can β-Defensin 2 Suspect the Dysbiosis of Urine Microbiota?

**DOI:** 10.3390/diagnostics11112082

**Published:** 2021-11-10

**Authors:** Sang Wook Lee, Si Hyun Kim, Kwang Woo Lee, Woong Bin Kim, Hae Woong Choi, Ji Eun Moon, Ahrim Moon, Young Ho Kim

**Affiliations:** 1 Department of Urology, Soonchunhyang University Bucheon Hospital, Soonchunhyang University College of Medicine, Bucheon 14584, Korea; bartol@schmc.ac.kr (S.W.L.); urolkw@schmc.ac.kr (K.W.L.); woongbins@schmc.ac.kr (W.B.K.); 2 Department of Urology, Soonchunhyang University Cheonan Hospital, Soonchunhyang University College of Medicine, Cheonan 31151, Korea; pigqueen0418@naver.com; 3 Divisions of Life Science, Korea University, Seoul 02841, Korea; haewoongchoi@korea.ac.kr; 4 Department of Biostatistics, Soonchunhyang University Hospital, Seoul 04401, Korea; moon6188@schmc.ac.kr; 5 Department of Pathology, Soonchunhyang University Bucheon Hospital, Soonchunhayng University College of Medicine, Bucheon 14584, Korea; armoon@schmc.ac.kr

**Keywords:** interstitial cystitis, biomarkers, urine specimen collection, antimicrobial peptide, beta-defensins

## Abstract

As urine is not sterile, inflammatory reactions caused by dysbiosis of the urinary microbiota may induce interstitial cystitis. A study was conducted to determine whether β-defensin 2 (BD-2), a specific antimicrobial peptide in the bladder, could be used as a novel diagnostic marker for ulcerative interstitial cystitis (IC). Urine samples from three female groups were examined: healthy controls (*n* = 34, Control group), non-Hunner type IC (*n* = 40, NHIC group), and Hunner type IC (*n* = 68, HIC group). Urine samples were collected via a transurethral catheter and assayed for BD-2 levels using enzyme linked immunosorbent assay. Under general or regional anesthesia, cystoscopy with diagnostic and therapeutic hydrodistension was performed in NHIC and HIC groups patients. These patients underwent a biopsy of the bladders. Based on the urinary specimens from 142 patients, BD-2 expression was found to be 18-fold higher in patients with Hunner type IC than in patients with non-Hunner type IC. The enhanced secretion of BD-2 exhibited a strong correlation with increased mast cell counts associated with bladder IC pathology. Enhanced urinary secretion of the antimicrobial peptide BD-2 from Hunner type IC patients associated with clinical phenotypes and demonstrated relatively robust levels to be used as a potential biomarker. Moreover, the increased urinary level of BD-2 may suggest a new possibility of biomarkers caused by dysbiosis of the urinary microbiota in ulcerative IC.

## 1. Introduction

Interstitial cystitis/bladder pain syndrome (IC/BPS) is traditionally defined as a chronic inflammatory disease with no definite cause of bacterial infection and is characterized by pain when the bladder is full and voiding; symptoms include changes in urination frequency and urgency [1]. This definition was established based on the assumption that urine is sterile when bacterial presence in the urinary tract was analyzed using conventional standard urine cultures. Recently, next-generation sequencing (NGS) has enabled the quantitative characterization of microbiomes, allowing for the discovery of hitherto uncultured bacteria in urine. Thereafter, there is consensus in the field that the urinary bladder is not a sterile environment [2,3].

Currently, the IC/BPS definition is in agreement with the Society of Urodynamics, Female Pelvic Medicine & Urogenital Reconstruction: “An unpleasant sensation (pain, pressure, and discomfort) perceived to be related to the urinary bladder, associated with lower urinary tract symptoms, persisting for more than six weeks in the absence of infection or other identifiable causes”. This criterion was chosen because it enables therapy to begin after a relatively short duration of symptoms, avoiding delay in treatment that might occur with longer symptom duration criteria (i.e., six months). Definitions used in research or clinical trials should be avoided in clinical practice, as many patients may be misdiagnosed, or diagnosis and treatment may be delayed if these criteria are used [4]. Many academic societies are making efforts to change the symptom period to a shorter duration of less than six weeks for early diagnosis and treatment. Until now, due to the definition, the issue of infection in the etiology of IC/BPS has been underestimated. The traditional idea is that active urinary tract infection (UTI) is excluded from IC/BPS diagnosis, and bacteria play a minimal role in IC/BPS. However, it is now understood that these traditional urine culture tools are inadequate for studying bacteria in patients with IC/BPS and presumed cystitis associated with chronic covert bacteria.

In a recent report on the urinary microbiome, Brubaker L & Multidisciplinary Approach to the Study of Chronic Pelvic Pain researchers reported that various bladder diseases (urinary incontinence, overactive bladder interstitial cystitis, etc.) could be attributed to the microbiome in the urinary bladder [5,6]. Although Nickel et al. emphasized that active UTI needs to be excluded from IC/BPS diagnosis and bacterial infection might be a very small cause of IC/BPS, it is now clear that the traditional urine culture techniques used to diagnose bacterial infection, not only in patients with IC/BPS but also in patients with presumed bacterial cystitis, fail to detect unculturable microbes that are potentially associated with these diseases. They reported the possible involvement of the urinary microbiome in IC/BPS through the assessment of culture-independent microbiological data analysis [7].

Unnatural changes in the microbiota composition, referred to as dysbiosis, can result in a variety of pathological conditions [8]. Dysbiosis increases the number of pathogenic bacteria in organs, potentially triggering the secretion of antimicrobial peptides (AMPs). AMPs are critical components of the innate immune system that serve as the first line of defense against pathogenic bacteria or dysbiosis in the urinary tract. Previous studies on bladder epithelial systems have supported the claim that AMP abundance in the epithelial microenvironment is associated with disease severity or inflammation [9,10]. Ali et al. reported that β-defensin 2 (BD-2), AMPs, and mast cell activator are persistently expressed in the bladder of women with IC/BPS [11]. BD-2 expression is induced in response to inflammation or infection and serves to augment epithelial barriers exposed to bacteria or inflammatory mediators [12]. Dysregulated homeostasis across mucosal sites can perturb microbial equilibrium and activate BD-2, acting as a sensor [13].

As a critical factor in a dysregulated chronic innate immune response comparable to that observed acutely during infection, BD-2 may be a principal factor directly associated with urothelial pathology in IC. Our study was designed with the possibility that some phenotypes of IC/BPS are the result of chronic inflammation associated with bacterial dysbiosis. We analyzed the levels of the diagnostic biomarker, BD-2, in patients with ulcerative IC/BPS in comparison to the normal healthy control group.

## 2. Materials and Methods

### 2.1. Patient Selection and Sample Collection

This study was a retrospective analysis of data obtained between January 2017 and January 2019 from a single tertiary medical facility. A total of 34 normal female patients with no symptoms related to urination and no abnormalities on urinalysis (Control group) were selected as the research subjects. Urine samples were collected via a transurethral catheter. Cystoscopy with diagnostic and therapeutic hydrodistension was performed in Patients with IC/BPS diagnostic criteria. According to the cystoscopic findings, 40 patients with the non-ulcerative type (non-Hunner type interstitial cystitis: NHIC group) and 68 patients with an ulcerative type (Hunner type interstitial cystitis: HIC group) were included in the study. Informed consent was obtained from all patients.

In NHIC and HIC groups, urine samples were collected using a transurethral catheter just before hydrodistension. In the NHIC group, a random biopsy was performed at least five times after hydrodistension. For the HIC group, a biopsy was performed at the site of the ulcer. The biopsied specimens obtained from the bladder lamina propria in NHIC and HIC groups were used to compare and analyze the number of mast cells. The specimens were immediately fixed in neutral buffered 10% formalin and sent to the Department of Pathology and analyzed by A Moon, a pathologist. Next, the specimens are stained by Hematoxylin/Eosin and toluidine blue. In addition, c-kit (CD117), immunohistochemistry stain, was also performed. The stained mast cells were counted by selecting four random fields per high-power field (HPF) under a light microscope (Nikon Eclipse E400, Tokyo, Japan, ×400; objective ×40; eyepiece ×10). The area per HPF is 0.25 mm^2^. Therefore, the area of 4 HPF is 1 mm^2^.

To accurately represent the chronic inflammation state, the stringent research criteria of the National Institute of Diabetes and Digestive and Kidney Diseases and the criteria for mast cell counts proposed by the European Society for the Study of Interstitial Cystitis were applied [14,15]. Patients with less than 28 mast cells/mm^2^ in ulcerative type (HIC group) were excluded from the study.

### 2.2. Urine Sample Processing

One milliliter urine sample was mixed with 10 µL of a 10-fold diluted protease inhibitor cocktail and stored at −80 °C. Urine samples stored in the biobank were held at room temperature and thawed before testing. The sample was centrifuged at 1500× *g* (3000 rpm) for 5 min to separate the debris, urine creatinine was determined in the supernatant, and BD-2 was tested in the same sample using enzyme-linked immunosorbent assay (ELISA) kits (Koma Biotech, Seoul, Korea).

### 2.3. Urine BD-2 Assay and Analyses

The concentration of BD-2 in urine was quantified by ELISA according to the manufacturer’s instructions (Koma Biotech, Seoul, Korea). The standard protein was prepared by stepwise dilution. A 200 µL aliquot of wash solution was added to each well and aspirated; the wells were washed three times with 300 µL of wash solution. Duplicate standards and patient samples were dispensed into a 96-well ELISA plate, and the plate was covered and incubated at room temperature for 2 h. The wells were emptied and washed four times, 100 µL of a detection antibody-diluted solution was added to each well, and the plate was incubated at room temperature for 2 h. We repeated the aspiration and washing process four times, and then 100 µL diluted horseradish peroxidase conjugate was added to each well and the plate was covered and incubated at room temperature for 30 min. The washing process was repeated four times, and then a 100 µL aliquot of substrate solution [3,3′,5,5′-Tetramethylbenzidine (TMB) or pink-ONE TMB] was added to each well, and the color was developed at room temperature. Finally, a 100 µL aliquot of 0.5 M stop solution was added. The optical density was determined using a microplate reader set to a wavelength of 450 nm.

### 2.4. Statistical Analyses

The Kruskal–Wallis test and the Mann–Whitney U test with Dunn’s multiple comparison post-hoc tests were performed to compare the groups. The ability of BD-2 to predict ulceration was measured and assessed. Logistic regression analyses were performed to define clinical cutoff values. The correlation between mast cell count and BD-2 was assessed using Spearman’s correlation analysis. All statistical analyses were performed using SPSS Statistics (version 26.0; IBM, Armonk, NY, USA) and R version 3.6.1 (The R Foundation for Statistical Computing, Vienna, Austria). A two-tailed *p* value of < 0.05, was considered significant.

## 3. Results

A total of 142 patients, including 34 patients aged 51.45 ± 13.01 years, 40 patients aged 55.38 ± 9.75 years, and 68 patients aged 57.89 ± 11.43 years, were assigned to Control, NHIC, and HIC groups, respectively. The symptom duration was 21.55 ± 9.64 months for NHIC group and 24.04 ± 7.95 months for HIC group (*p* > 0.05). The number of bladder ulcers in HIC group diagnosed with cystoscopy were 3.42 ± 0.98 (Table 1).

**Figure 1:**
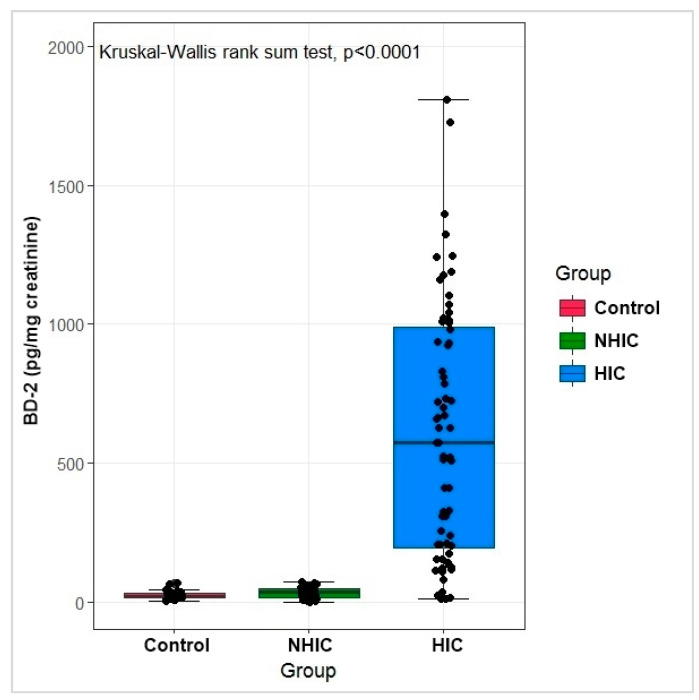
BD-2 level in all patients group. (BD-2: β-defensin 2, NHIC: non-Hunner type interstial cystitis, HIC: Hunner type interstial cystitis).

**Figure 2:**
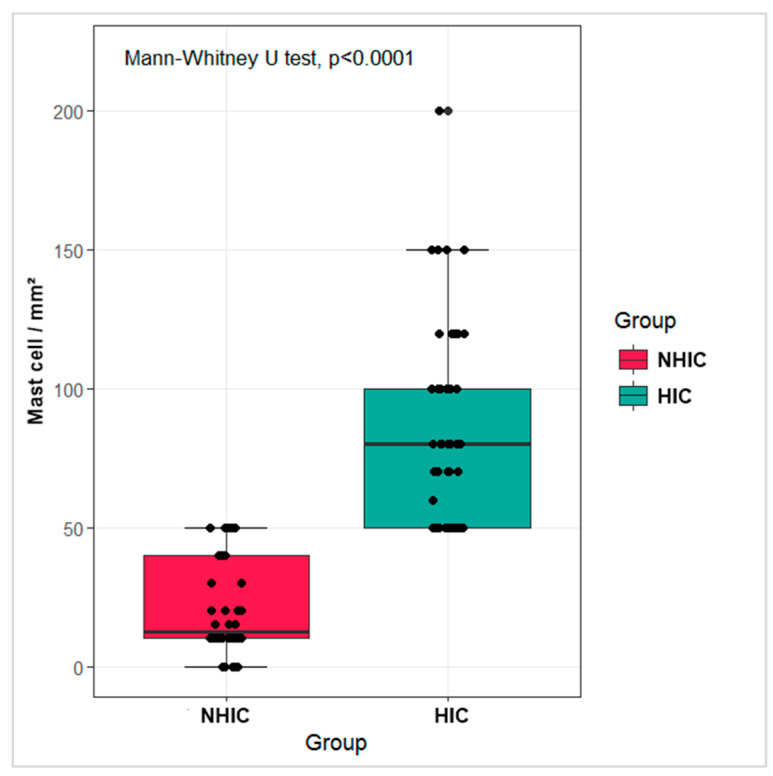
Mast cell count in interstitial cystitis group. (NHIC: non-Hunner type interstial cystitis, HIC: Hunner type interstial cystitis).

**Figure 3:**
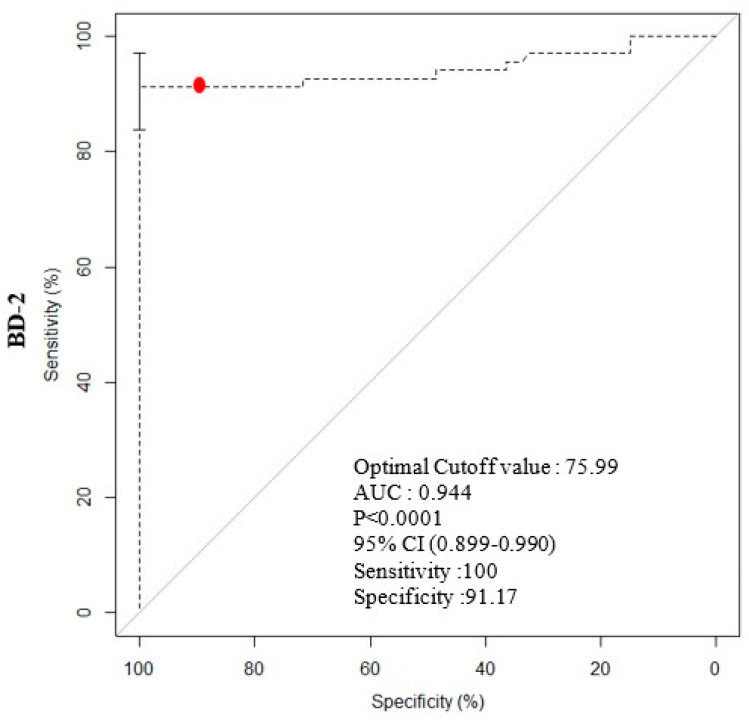
Logistic regression analysis for Urine BD-2 concentration (pg/mg); with optimal cut off value(the red dot). Area under the curve (AUC) presented with confidence intervals. Most appropriate cut-off point displayed along with sensitivity and specificity. (BD-2: β-defensin 2).

The median BD-2 (pg/mg creatinine) was 20.05 pg/mg (range: 13.22–29.56) in Control group, 32.98 pg/mg (range: 15.02–48.70) in NHIC group, and 574.98 pg/mg (range: 194.98–988.65) in HIC group (*p* < 0.01; Table 2, Figure 1). No significant difference was detected between Control and NHIC groups, but significant differences were observed between Control and HIC groups and between NHIC and HIC group. The median mast cell count was 20.00 cells/mm^2^ (range: 15.00–40.0) in NHIC group and 80.00 cells/mm^2^ (range: 50.00–100.00) in HIC group (*p* < 0.05; Table 2, Figure 2). The cutoff value from the logistic regression analyses was 75.99 pg/mg for BD-2 (sensitivity: 100%, specificity: 91.17%; Figure 3).

A correlation analysis was performed to determine the correlation between the mast cell count and the concentration of BD-2; the correlation between mast cell count and BD-2 concentration was 0.635 (*p* < 0.0001) in NHIC group and 0.9214 (*p* < 0.0001) in HIC group. The combination of NHIC and HIC groups yielded 0.9096 (*p* < 0.0001). These results confirm that the number of mast cells increased and was positively correlated with increasing BD-2 concentration associated with bladder pathology, such as non-ulcerative or ulcerative IC (Table 3).

## 4. Discussion

IC is a heterogeneous disease of unknown etiology. The two main etiologic theories for IC are a defect in the bladder cytoprotection and an increased number of mast cells in the bladder [16]. The increased mast cells are activated by cytokines, bacterial and viral superantigens, immunoglobulin aggregates, neuropeptides, acetylcholine, and neurotensin. Activated mast cells degranulate to secrete histamine, kinins, proteases, cytokines, leukotrienes, prostaglandins, and nitric oxide [17,18]. In ulcerative IC, mast cells in the submucosa and detrusor muscles are increased [19]. Mast cells are known to increase 10-fold in ulcerative IC and 2-fold in non-ulcerative IC, and similar results were reported in this study [20]. Many studies in humans with IC and in animal models of cystitis strongly suggest that bladder mast cell activation is a major component of the pathophysiology of ulcer and non-ulcer IC. However, studies have focused on neuroinflammation or toxic inflammation rather than an association with mastocytosis of microbiota origin. Our study identified a novel biomarker suitable for these two major theories.

Various inflammatory diseases (e.g., irritable bowel syndrome, inflammatory bowel disease, Crohn’s disease, etc.) that occur in the intestine can occur in the bladder (e.g., overactive bladder, urge incontinence, and IC/BPS). According to a report on Crohn’s disease (CD) [21], sufficient evidence suggesting a single bacterium contributing to CD pathogenesis is unavailable. Alternatively, dysbiosis or bacterial imbalance is more widely accepted as a leading factor in disrupted host–immune system cross-talk, resulting in subsequent intestinal inflammation. CD is associated with damage to the glycosaminoglycan (GAG) layer, which appears to exhibit histopathological characteristics similar to those of IC. This suggests a new possibility that the immune inflammatory reaction is caused by dysbiosis of the microbiota in the bladder rather than simply causing IC/BPS due to infection by specific bacteria [7,22,23].

During bacterial cystitis, potentially caused by microbial dysbiosis, the uropathogenic *Escherichia coli* activates the innate immune responses in which the superficial bladder epithelium can secrete danger signals when stressed. For example, during infections, stressed host epithelial cells release the alarmin interleukin (IL)-33, which can activate innate immune cells [24]. Another class of alarmins secreted by infected superficial bladder epithelial cells are defensins [25]. These are potent cationic peptides that exhibit antibacterial activity and are potent mast cell activators [26,27]. Activated mast cells expedite the exfoliation of infected urothelial cells to reduce the bacterial load in infected bladders [28]. Our study demonstrated that patients with ulcerative IC produced robustly higher mast cell counts and urinary BD-2 than that produced by patients with non-ulcerative IC. A strong association was found between the BD-2 concentration and IC. Therefore, the interaction between mast cells and AMPs may be critical for elucidating the pathogenesis of ulcerative IC. Yoshiyuki et al. reported that there was no significant difference in mast cell density between the IC group and the non-IC group. However, there was also a difference in the mast cell density in the lamina propria area between the HIC and NHIC groups [29].

Microbiota in the bladder and the severity of symptoms are reported to be associated with urine antimicrobial peptides. Host AMPs and resident bacterial communities (microbiota) are both critical components of normal host innate immunity that help to prevent infection and pathogen-induced inflammation. The role of AMPs is important not only in UTI, but also in immune responses that cause chronic inflammation [12].

Defensins are divided into alpha, beta, and theta groups depending on the location of cysteine and the disulfide bridges. Unlike α-defensin, which is produced by neutrophils, β-defensin is commonly found in urothelial cells; six β-defensins have been identified in human tissue. BD-2 is produced after exposure to pro-inflammatory cytokines and bacteria [12,30]. Prasad et al. reported the complex role of host defense peptides in the pathophysiology of several immune-mediated inflammatory diseases. They suggested five inflammatory responses of AMPs, a potent immune modulator, (1) neutralization of bacterial toxins, (2) chemoattraction and activation of immune cells, (3) initiation of adaptive immunity, (4) neovascularization and wound healing, and (5) anti- or pro-tumor activity [31].

As AMPs interact with immunological receptors on both the innate and adaptive forms of the immune system, such as pattern recognition receptors, damage, or chemokine receptors, inflammasomes, and associated complement systems, they help to bridge the gap between innate and adaptive immunity [31,32,33,34]. Thus, AMPs, particularly BD-2, may increase as a result of the abnormal immune response and defense mechanism against inflammation in ulcerative IC with damage to the GAG layer. If the bladder microbiota causes chronic inflammation and ulcerative disease, similar to that caused by intestinal microbiota, the levels of AMP can increase in some patients with IC/BPS. Defensins are small cationic AMPs that suppress the inflammatory response through direct antibacterial action and regulation of the cellular immune response. BD-2 causes chemotaxis of dendritic cells and memory T cells by interacting with chemokine receptors, connecting innate immune responses, and acquired adaptive immune responses. Additionally, the secretion level of BD-2 can be enhanced to perform a broad spectrum of antibacterial, antiviral, and antifungal actions when bladder epithelial cells are stimulated [35]. This effect indicated that GAGs stimulate the biosynthesis of antibacterial peptides, suggesting the ability of bladder cells to secrete host defense peptides [36].

Our study demonstrated the possibility of using urinary BD-2 level as a novel biomarker for ulcerative interstitial cystitis. However, this study had the following limitations. First, this was a retrospective, single-center study. Second, the mast cell count and urinary BD-2 levels may have been affected by comorbidities, which was unaccounted for in this study. Furthermore, mast cell count was not performed in the control group. Unexpected systemic bias could have resulted in different outcomes in various patient groups. Nevertheless, the results of our study need to be confirmed and validated using a prospective, large-scale, multicenter study. Third, as in the status of the urinary microbiome, comparative studies cannot be conducted, so only indirect suggestions using AMPs related to microbiota can be performed. This is because it is difficult to collect urine from patients with interstitial cystitis due to low bladder capacity. Finally, it is challenging to measure both BD-2 and the microbiome at the same time because urine in the bladder has low biomass. A further well-designed study, such as urine DNA NGS, is needed to overcome these limitations.

## 5. Conclusions

Urinary BD-2 level, as a novel biomarker, was higher in the HIC group than in the normal and NHIC groups. The cut-off value of BD-2 concentration is high at 75.99 pg/mg (sensitivity: 100%, specificity: 91.17%). Thus, if a patient with bladder pain and absence of bacteria had a BD-2 level of 75.99 pg/mg or more, this suggests that an accurate diagnosis may be necessary with cystoscopy. The increased urinary level of the AMP BD-2 may indicate chronic bladder inflammation caused by dysbiosis of the urinary microbiota in ulcerative interstitial cystitis.

## Figures and Tables

**Table 1:** Baseline characteristics per group.

Variable	Control Group (*n* = 34)	NHIC Group (*n* = 40)	HIC Group (*n* = 68)	Comparision (*p*-Value)
Age (years)	51.45 ± 13.01	55.38 ± 9.75	57.89 ± 11.43	0.652
Symptom duration (months)		21.55 ± 9.64	24.04 ± 7.95	0.228
Ulcers			3.42 ± 0.98	

Data was presented as mean ± standard deviation (SD) for age and symptom duration. *p*-values were derived from ANOVA test and independent T-test. IC: interstitial cystitis, NHIC: non—Hunner type IC, HIC: Hunner type IC.

**Table 2:** Comparison of BD-2 and Mast cell count in Control group, Non-ulcer, and Ulcer group.

Variable	Control(1)	NHIC(2)	HIC(3)	*p*-Value	Post hoc Analysis(Multiple Comparison)		
	(*n* = 34)	(*n* = 40)	(*n* = 68)		1 vs. 2	1 vs. 3	2 vs. 3
BD-2 (pg/mg creatinine)	20.05 (13.22,29.56)	32.98 (15.02,48.70)	574.98 (194.98,988.65)	<0.0001	0.335	<0.0001	<0.0001
Mast cell (cell/mm^2^)		12.50 (10.00,40.00)	80.00 (50.00,100.00)	<0.0001			

*p*-values were calculated by Kruskal-Wallis test for and Mann-whitney U test for continuous variables. Data is presented as a Median (Q1,Q3) for continuous variables. Post-hoc analysis with Dunn’s multiple comparison method was performed to compare between each groups. BD-2: β-defensin 2, NHIC: non-Hunner type interstitial cystitis, HIC: Hunner type interstitial cystitis.

**Table 3:** Correlation analysis between Mast cell count and BD-2 levels.

		Mast Cell	BD-2
NHIC group	Correlation coefficient (r)	1	0.635
	Significance (2-tailed)	.	<0.0001
	N	40	40
HIC group	Correlation coefficient (r)	1	0.9214
	Significance (2-tailed)	.	<0.0001
	N	68	68
NHIC and HIC group	Correlation coefficient (r)	1	0.9096
	Significance (2-tailed)		<0.0001
	N	108	108

r: Spearman correlation coefficient. BD-2: β-defensin 2, NHIC: non-Hunner type interstitial cystitis, HIC: Hunner type interstitial cystitis.
